# Aldosterone Induced Galectin-3 Secretion In Vitro and In Vivo: From Cells to Humans

**DOI:** 10.1371/journal.pone.0095254

**Published:** 2014-09-02

**Authors:** Yen-Hung Lin, Chia-Hung Chou, Xue-Ming Wu, Yi-Yao Chang, Chi-Sheng Hung, Ying-Hsien Chen, Yu-Lin Tzeng, Vin-Cent Wu, Yi-Lwun Ho, Fon-Jou Hsieh, Kwan-Dun Wu, the TAIPAI Study Group

**Affiliations:** 1 Department of Internal Medicine, National Taiwan University Hospital and National Taiwan University College of Medicine, Taipei, Taiwan; 2 Department of Obstetrics and Gynecology, National Taiwan University Hospital and National Taiwan University College of Medicine, Taipei, Taiwan; 3 Department of Internal Medicine, Taoyuan General Hospital, Taoyuan, Taiwan; 4 Department of Cardiology, Cardiovascular Center, Far Eastern Memorial Hospital, New Taipei City, Taiwan; Institute of Hepatology, Foundation for Liver Research, United Kingdom

## Abstract

**Context:**

Patients with primary aldosteronism are associated with increased myocardial fibrosis. Galectin-3 is one of the most important mediators between macrophage activation and myocardial fibrosis.

**Objective:**

To investigate whether aldosterone induces galectin-3 secretion in vitro and in vivo.

**Methods and Results:**

We investigated the possible molecular mechanism of aldosterone-induced galectin-3 secretion in macrophage cell lines (THP-1 and RAW 264.7 cells). Aldosterone induced galectin-3 secretion through mineralocorticoid receptors via the PI3K/Akt and NF-κB transcription signaling pathways. In addition, aldosterone-induced galectin-3 expression enhanced fibrosis-related factor expression in fibroblasts. We observed that galectin-3 mRNA from peripheral blood mononuclear cells and serum galectin-3 levels were both significantly increased in mice implanted with aldosterone pellets on days 7 and 14. We then conducted a prospective preliminary clinical study to investigate the association between aldosterone and galectin-3. Patients with aldosterone-producing adenoma had a significantly higher plasma galectin-3 level than patients with essential hypertension. One year after adrenalectomy, the plasma galectin-3 level had decreased significantly in the patients with aldosterone-producing adenoma.

**Conclusion:**

This study demonstrated that aldosterone could induce galectin-3 secretion in vitro and in vivo.

## Introduction

An excess of aldosterone plays a pivotal role in the development and progression of cardiac diseases such as primary aldosteronism (PA) and heart failure (HF)[1], [2]. PA affects 5–13% of patients with hypertension, and is characterized by an inappropriate production of aldosterone[1]. Increasing evidence indicates that PA is much more prevalent than previously believed, and that this disease is the most frequent cause of secondary hypertension[1]. Using aldosterone blockade with spironolactone has been shown to improve the outcome of HF patients[3], and is now the standard therapy for severe HF.

Aldosterone has been found to induce myocardial fibrosis in the cardiovascular system. In an animal study, aldosterone infusion with high salt intake induced cardiac hypertrophy and myocardial interstitial fibrosis and scarring in the right and left ventricles[4]. In clinical studies, patients with PA have been found to have more prominent left ventricular (LV) mass and cardiac fibrosis than patients with essential hypertension (EH), which may be due to the high plasma aldosterone levels in these patients[5], [6]. Unilateral aldosterone-producing adenoma (APA) is the most common subtype of PA; it can be cured by adrenalectomy after which the increased LV mass and myocardial fibrosis regress[5], [7]–[10].

Despite the direct effect of aldosterone to induce fibrosis, aldosterone also induces macrophage activation and low grade inflammation, which may play an important role in cardiac fibrosis and myocardial dysfunction[2]. Deletion of mineralocorticoid receptors (MR) from macrophages has been shown to protect against deoxycorticosterone/salt-induced cardiac fibrosis, which implies that macrophages play an important role in aldosterone-induced cardiac fibrosis[11]. However, the mediators of aldosterone through inflammation to fibrosis are still unclear. Among a number of candidate mediators, galectin-3 was found to be one of the most likely candidates in several animal[12], [13] and clinical studies[14], [15]. Galectin-3 is a β-galactoside-binding animal lectin which is highly expressed and secreted by macrophages[16]. Galectin-3 has been shown to be involved in fibrosis in various organs, including the lungs[17], liver[18], kidneys[19], and heart[12]. In an animal model of renal fibrosis, knocking-out the galectin-3 gene was found to decrease renal fibrosis without changing macrophage recruitment or macrophage proinflammatory cytokine profiles[19]. In addition, the adoptive transfer of wild-type macrophages into galectin-3 knocked-out mice was found to restore the fibrotic phenotype in kidneys, suggesting that galectin-3 expression and secretion is a key mechanism linking macrophages to myofibroblast activation and tissue fibrosis[19]. A recent study further showed that aldosterone increased vascular galectin-3 expression, inflammation, and collagen formation in wild-type mice, whereas no changes occurred in galectin-3 knocked-out mice[20].

We hypothesized that aldosterone plays an important role in galectin-3 production in macrophages, which may further cause fibrosis in the cardiovascular system. Therefore, we conducted this study to evaluate galectin-3 secretion by aldosterone stimulation in vitro and vivo.

## Materials and Methods

### Cell cultures of THP-1, Raw 264.7, human fibroblast 293T and mouse fibroblast NIH/3T3 cell lines

THP-1, RAW 264.7, 293T and NIH/3T3 cell lines were obtained from the American Type Culture Collection (Rockville, MD, USA). THP-1 and RAW 264.7 cell lines were maintained in RPMI 1640 medium supplemented with 2 mmol/L glutamine, 100 U/mL penicillin, 100 µg/mL streptomycin to inhibit bacterial contamination, and 10% FCS for cell growth. The human fibroblast 293T and mouse fibroblast NIH/3T3 cell lines were cultured in DMEM containing 10% FBS, 100 U/mL penicillin, and 100 µg/mL streptomycin to inhibit bacterial contamination.

### Concentration of aldosterone-induced galectin-3 secretion

THP-1 and RAW 264.7 cells (10^6^/ml) were treated with different doses of aldosterone (10^−9^, 10^−8^, 10^−7^, 10^−6^, 10^−5^ M). After 24 hours, the cell culture supernatants were collected for galectin-3 detection.

### Timing of aldosterone-induced galectin-3 secretion

THP-1 and RAW 264.7 cells (10^6^/ml) were treated with aldosterone (10^−6^ M), for 0, 6, 12, 18, 24, 30, 36, 42, and 48 hours. The cell culture supernatants were then collected for galectin-3 analysis.

### Detection of galectin-3 level by enzyme immunoassay

The level of galectin-3 in the cell culture supernatant was determined using a commercially available enzyme immunoassay (EIA) kit (R&D Systems) according to the manufacturer's instructions. The catalog numbers for human galectin-3 and mouse galectin-3 were DGAL30 and DY1197, respectively. Each measurement was performed in triplicate, and the average value was then recorded as ng/mL.

### Aldosterone induced galectin-3 secretion through mineralocorticoid or glucocorticoid receptors

THP-1 and RAW 264.7 cells (10^6^/ml) were pretreated with 10^−7^ M of spironolactone (Sigma, St. Louis, MO, USA; dissolved in ethanol) or 10^−7^ M RU-486 (Sigma, St. Louis, MO, USA; dissolved in DMSO) 1 hour prior to aldosterone (10^−6^ M) treatment. After 24 hours, the cell culture supernatants were collected for galectin-3 detection by EIA.

### Real-time quantitative reverse transcription polymerase chain reaction

Glyceraldehyde-3-phosphate dehydrogenase (GAPDH), galectin-3, fibronectin, and procollagen (I) mRNA expressions were measured using a fluorescein quantitative real-time PCR detection system (Light Cycler DNA Master SYBR Green I; Roche Molecular Biochemicals, Indianapolis, IN). The primer pairs were as follows: GAPDH, 5′-GGG AAG GTG AAG GTC GG-3′ and 5′-TGG ACT CCA CGA CGT ACT CAG-3′; galectin-3, 5′-GGCCACTGATTGTGCCTTAT and 5′-TCTTTCTTCCCTTCCCCAGT [21]; mouse galectin-3, 5′-AGC TTA TCC TGG CTC AAC TG and 5′-CGG AGG TTC TTC ATC CGA TG; human fibronectin, 5′-CAG GAT CAC TTA CGG AGA AAC AG-3′ and 5′-GCC AGT GAC AGC ATA CAC AGT G-3′ [22], mouse fibronectin forward-5′-GTCAGTGTCTCCAGTGTCTAC-3′ and 5′-TGGCTTGCTGGCCAATCAGT-3′, human procollagen (I), 5′-CTC GAG GTG GAC ACC ACC CT-3′ and 5′-CAG CTG GAT GGC CAC ATC GG-3′) [23], mouse collagen (I) 5′-TGTTCGTGGTTCTCAGGGTAG-3′ and 5′-TTGTCGTAGCAGGGTTCTTTC-3′. The amplification program consisted of one cycle of initial incubation at 61°C for 20 minute, followed by 50 cycles of denaturation at 95°C for 10 seconds, annealing at 55–57°C for 10 seconds, and extension at 72°C for 10 seconds. The amount of indicated mRNA was normalized by that of GAPDH mRNA and presented in arbitrary units, with 1 U corresponding to the value in the cells treated with the vehicle control.

### Down-stream pathway

THP-1 and RAW 264.7 cells (106/ml) were pretreated with the indicated inhibitors 1 hour prior to aldosterone (10^−6^ M) treatment. After 24 hours, the cell culture supernatants were collected for galectin-3 detection by EIA. The inhibitors were PD98059 (Sigma, St. Louis, MO, USA; ERK inhibitor), LY294002 (Sigma, St. Louis, MO, USA; phosphoinositide 3-kinase inhibitor), SB203580 (Sigma, St. Louis, MO, USA; p38 inhibitor), Ro318220 Sigma, St. Louis, MO, USA; protein kinase C inhibitor), and SP600125 (Sigma, St. Louis, MO, USA; Jun N-terminal kinase inhibitor).

For nuclear factor (NF)-κB pathway analysis, THP-1 and RAW-264.7 cells were serum-starved for 24 hours and then pre-treated with 10^−7^ M of spironolactone, LY294002 or BAY117082 (Sigma, St. Louis, MO, USA; NF-κB inhibitors) for 1 hour before 10^−6^ M aldosterone treatment. Nuclear and cytosolic proteins were collected after 1 hour, and the level of the NF-κB p65 subunit was determined by Western blotting.

### Synthesis of NF-κB decoy oligodeoxynucleotides

We used synthetic double-stranded decoy oligodeoxynucleotide (ODN) cis elements to block the binding of nuclear factors to the promoter regions of targeted genes. The following conserved sequences of the phosphorothioate ODN between humans and mice were used: NF-κB decoy ODN: 5′-CCTTGAAGGGATTTCCCTCC-3′ and 3′-GGAACTTCCCTAAAGGGAGG-5′; AP-1 decoy ODN: 5′-TGTCTGACTCATGTC-3′ and 3′-CAGACTGAGTACA-5′; scrambled decoy ODN: 5′-TTGCCGTACCTGACTTAGCC-3′ and 3′-AACGGCATGGACTGAATCGG-5′. The ODNs were mixed with an equal volume (10∶1) of TransFast for 15 minutes, and then incubated with the cells in a serum-free medium.

### Preparation of conditioned medium

Cultures of the THP-1 and RAW 264.7 cells (2×10^6^/10-mm dish) were rinsed twice with PBS and then cultured in 5 ml of serum-free RPMI 1640 with aldosterone (10^−6^ M) or vehicle treatment for 24 hours. The conditioned medium was collected and then clarified by centrifugation (4°C, 10,000 rpm, 5 minutes) to remove cell debris. A final solution of 25 mM HEPES buffer (pH 7.4), 1 mg ml^−1^ leupeptin, 1 mM phenylmethylsulfonyl fluoride, 1 mM EDTA, 0.02% NaN_3_, and 0.1% BSA (Sigma) was the added. The conditioned medium was then frozen and stored at −70°C until use.

### RNA interference

Small interfering RNA (siRNA) duplexes were purchased from Santa Cruz Biotechnology (Santa Cruz, CA, USA). The targeted siRNAs for human galectin-3, mouse galectin-3, and control siRNA were sc-155994, sc-35443 and sc-37007, respectively. The THP-1 and Raw 264.7 cells were transfected with siRNA at a concentration of 25 nM in serum-free Opti-MEM using the Oligofectamine method (Invitrogen, Carlsbad, CA, USA).

### Cell growth determined by MTT assay

Human fibroblast 293T and mouse fibroblast NIH/3T3 cell lines were plated onto 96-well microplates at a density of 5×10^3^ cells/well with the conditioned media for cell growth assay. Briefly, the cells were cultured at 37°C for the indicated times, and 30 µl of MTT solution (5 mg/ml) was then added into each well followed by incubation for 4 hours in darkness. Formazan grains were dissolved in DMSO, and the absorbance at 570 nm was read using an ELISA plate reader.

### Aldosterone release pellet implanted mouse model

Six-week-old ICR wild-type male mice were obtained from the Animal Center of the Medical College of National Taiwan University and kept in standard animal housing conditions under the guidelines for the care and use of experimental animals. The experiments were approved by the Animal Care and Use Committee of the Medical College of National Taiwan University. Mice with a similar body weight (25–30 g) were randomly divided into two treatment groups (vehicle and aldosterone; N = 5 per group). The mice were implanted subcutaneously with a 20-d continuous aldosterone release pellet (Innovative Research of America, Sarasota, FL). The vehicle control mice received a placebo pellet. Mice whole blood was collected on days 0, 7, and 14. The sera used for galectin-3 and total RNA were purified from peripheral blood mononuclear cells and used for galectin-3 mRNA analysis.

### Patients

Seventeen patients with essential hypertension (EH) and 7 patients with APA who did not take spironolactone within the previous 6 months and who were prepared to receive adrenalectomy were enrolled. The patients were evaluated and registered in the Taiwan Primary Aldosteronism Investigation (TAIPAI) database. This database was established for quality assurance in one medical center (National Taiwan University Hospital, Taipei, Taiwan) and its three branch hospitals in different cities around Taiwan (National Taiwan University Hospital Yun-Lin branch, Yun-Lin, southern Taiwan; Far-Eastern Memorial Hospital, Taipei, northern Taiwan; and Tao-Yuan General Hospital, Tao-Yuan, central Taiwan) [24], [25]. The medical history of each patient including demographic and medication data was carefully recorded. Serum biochemistry studies of these patients were performed at the initial evaluation at National Taiwan University Hospital. Plasma aldosterone concentration (PAC) was measured by radioimmunoassay using a commercial kit (Aldosterone Maia Kit; Adaltis Italia, Bologna, Italy), and plasma renin activity (PRA) was measured as the generation of angiotensin-I in vitro using a commercially available radioimmunoassay kit (Cisbio, Bedford, MA). This study was approved by the Institutional Review Board of National Taiwan University Hospital, and all subjects provided written informed consent, including for the storage of their information in the hospital database and usage for research.

### Diagnosis of APA

The diagnosis of APA was established when all of the following “modified 4 corners score” criteria were met: (1) evidence of autonomous excess aldosterone production based on an aldosterone-renin ratio (ARR) >35 or urine ≥12 µg/24 hours, and a TAIPAI score of more than 60% [26] and post-saline loading PAC >10 ng/dl; (2) lateralization of aldosterone secretion in adrenal vein sampling or during dexamethasone suppression NP-59 SPECT/computed tomography[27]; (3) evidence of adenoma in computed tomography scans; and (4) pathologically proven adenoma after adrenalectomy if the patient received surgery, and cure of hypertension without anti-hypertensive agents or improved hypertension, potassium, PAC, and PRA as described.

### Plasma galectin-3 analysis

Venous blood samples were collected in serum separation tubes after overnight fasting. After clotting and centrifugation, the serum was stored at −60 °C until analysis. Galectin-3 was measured by an ELISA kit (Bender Medsystems, Vienna, Austria) and measured on a Victor 2 plate reader (Perkin Elmer, Turku, Finland). Calibration of the assay was performed according to the manufacturer's protocol. Values were normalized to a standard curve. The intra- and inter-assay variances for galectin-3 were 5.6% and 8.6%, respectively.

### Post-adrenalectomy follow-up

Clinical evaluation and plasma galectin-3 level measurements were performed 1 year after adrenalectomy. Hypertension was considered cured if the blood pressure decreased to 140/90 mm Hg or less after adrenalectomy, and anti-hypertensive medications were not required[28] one year post adrenalectomy [8]. The patients who were cured within one year but later developed hypertension were still classified as being cured.

### Statistical analysis

In the cell study, each experiment was performed in triplicate and all experiments were repeated at least three times on different occasions. A t test was used to evaluate statistically significant differences between the aldosterone treatment and vehicle control groups in the specified tests. In the human study, data were expressed as mean ±SD. Comparisons for continuous data between the PA and EH patients were made using a t test. Differences between proportions were assessed by the chi-square or Fisher exact test. Comparisons between pre-operative and post-operative parameters were made using a paired t test. Pearson's correlation test was used to analyze the association between two parameters. Data of PAC, PRA, and ARR were log-transformed before the correlation study due to the non-normality which was determined by the Kolmogorov-Smirnov test. Before further analysis, the log-transformed data were tested again to assure the normality of distribution. A *p* value less than 0.05 was selected as the statistically significant cutoff value. A *p* value between 0.05 and 0.1 was considered to indicate borderline significance.

## Results

### Concentration and timing of aldosterone-induced galectin-3 secretion

**Figure 1:**
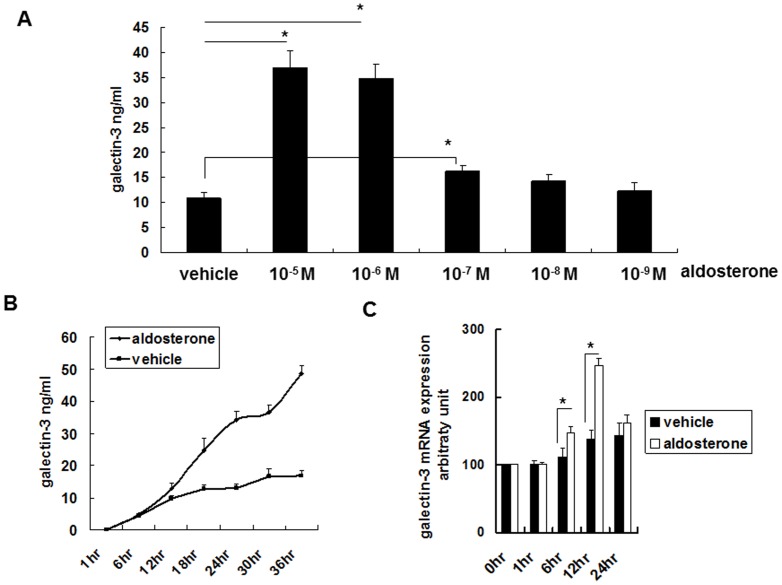
Aldosterone induced galectin-3 expression in human monocyte cell line THP-1. (A) Aldosterone induced galectin-3 expression dose dependently. THP-1 cells (1×10^6^ cells/ml) were serum-starved for 24 h prior to 24-h aldosterone treatment with various concentrations as indicated. (B) Aldosterone induced galectin-3 protein expression time dependently. THP-1 cells (1×10^6^ cells/ml) were serum-starved for 24 h prior to 10^−6^ M aldosterone or vehicle treatment for various durations. (C) Aldosterone transcriptionally regulated galectin-3 expression. THP-1 cells (1×10^6^ cells/ml) were serum-starved for 24 h prior to 10^−6^ M aldosterone or vehicle treatment for various durations. ^*^ *P*<0.05.

**Figure 2:**
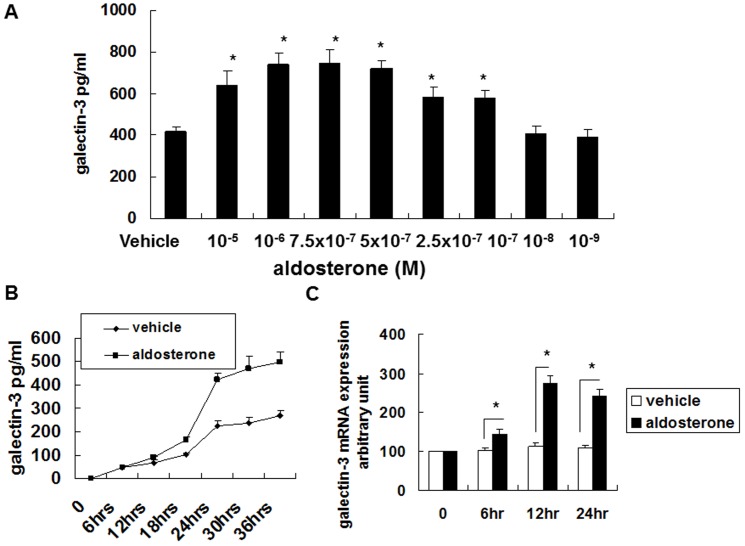
Aldosterone induced galectin-3 expression in mouse macrophage cell line Raw 264.7. (A) Aldosterone induced galectin-3 expression dose dependently. Raw 264.7 cells (1×10^6^ cells/ml) were serum-starved for 24 h prior to 24-h aldosterone treatment with various concentrations as indicated. (B) Aldosterone induced galectin-3 protein expression time dependently. Raw 264.7 cells (1×10^6^ cells/ml) were serum-starved for 24 h prior to 10^−6^ M aldosterone or vehicle treatment for various durations. (C) Aldosterone transcriptionally regulated galectin-3 expression. Raw 264.7 cells (1×10^6^ cells/ml) were serum-starved for 24 h prior to 10^−6^ M aldosterone or vehicle treatment for various durations. ^*^ *P*<0.05.

Galectin-3 was synthesized in the cultured THP-1 and RAW 264.7 cells with aldosterone stimulation in a dose-dependent manner (Figure 1A, 2A). Aldosterone 10^−5^ to 10^−7^ M significantly induced galectin-3 secretion. After 12 hours of aldosterone (10^−6^ M) stimulation, galectin-3 secretion in the cultured THP-1 and RAW 264.7 cells was significantly increased (Figure 1B, 2B).

### Galectin-3 mRNA analysis

Aldosterone 10^−6^ M significantly induced galectin-3 mRNA expression in the cultured THP-1 and RAW-264.7 cells (Figure 1C, 2C). This indicated that the changes in the expression of galectin-3 after aldosterone stimulation were through transcriptional regulation.

### Signal transduction pathway

**Figure 3:**
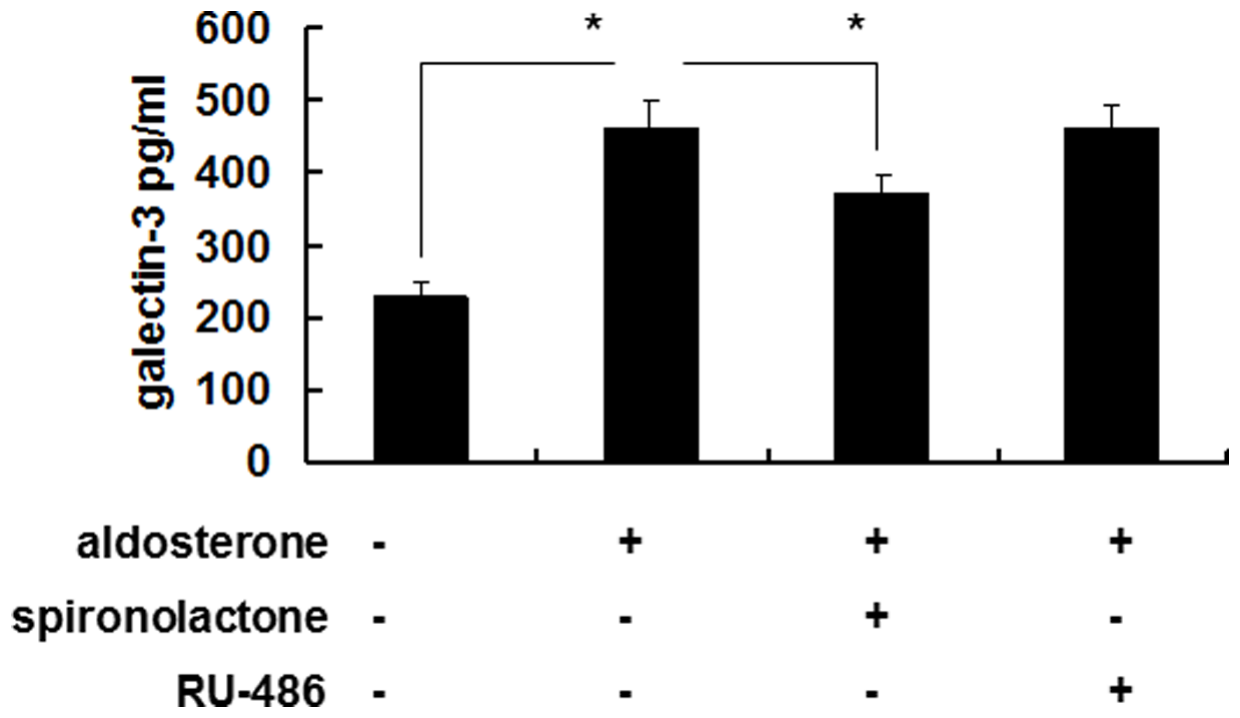
Aldosterone induced galectin-3 expression mainly through MRs in human monocyte cell line THP-1. THP-1 cells (1×10^6^ cells/ml) were serum-starved for 24 h, and then treated with 10^−7^ M of spironolactone or 10^−7^ M of RU-486 for 1 h followed by 10^−6^ M aldosterone or vehicle treatment for 24 h. The cell culture supernatants were then collected for galectin-3 detection by enzyme immunoassay. Comparison is between the indicated groups. *P<0.05. MR =  mineralocorticoid receptor.

**Figure 4:**
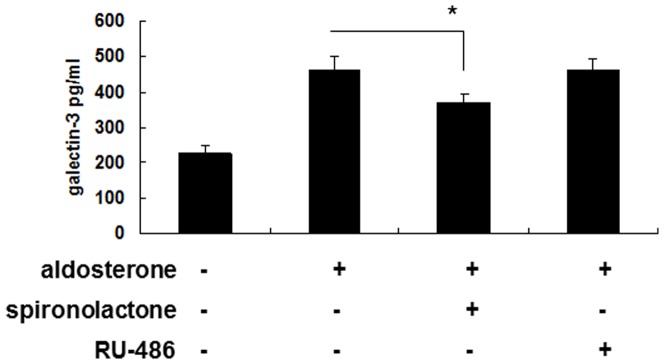
Aldosterone induced galectin-3 expression mainly through MRs in macrophage cell line Raw 264.7. Raw 264.7 cells(1×10^6^/ml) were serum-starved for 24 h, then treated with 10^−7^ M of spironolactone or 10^−7^ M of RU-486 for 1 h, followed by 10^−6^ M aldosterone or vehicle treatment for 24 h. The cell culture supernatants were then collected for galectin-3 detection by enzyme immunoassay. *P<0.05. MR =  mineralocorticoid receptor

The secretion of galectin-3 after aldosterone stimulation was inhibited by pretreated with 10^−7^ M spironolactone, but not by 10^−7^ M RU-486 (Figure 3, 4). This suggested that the secretion of galectin-3 was via mineralocorticoid receptors (MRs) rather than glucocorticoid receptors.

**Figure 5:**
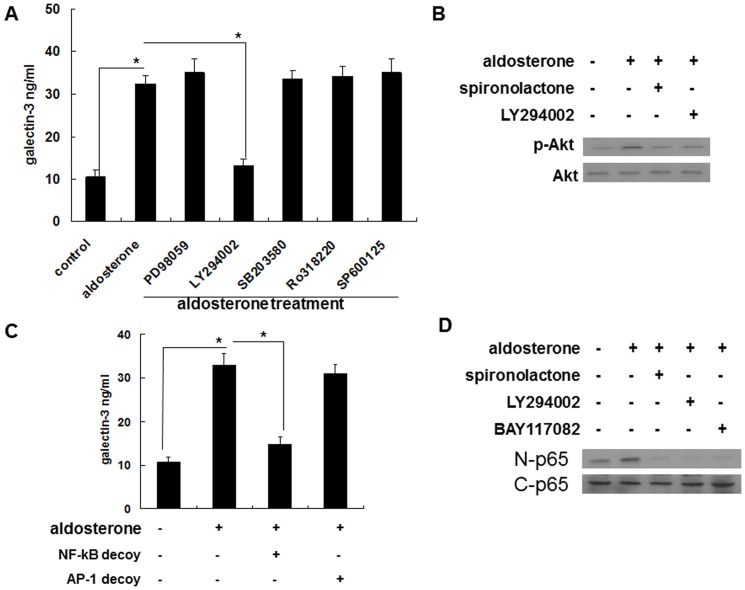
Aldosterone induced galectin-3 expression in THP-1 cells via a MR/PI3K/Akt/NF-κB signaling pathway. (A) Aldosterone induced galectin-3 expression in THP-1 cells via the PI3K/Akt signaling pathway. THP-1 cells were serum-starved for 24 h and then pre-treated with PD98059 (50 µg/ml), LY294002 (50 µg/ml), SB203580 (5 µg/ml), Ro318220 (5 nM), and SP600125 (10 µM) for 1 h prior to 10^−6^ M aldosterone or vehicle treatment for 24 h. (B) Aldosterone induced Akt phosphorylation via MR-mediated signaling. THP-1 cells were serum-starved for 24 h, then pre-treated with different chemical inhibitors for 1 h prior to 10^−6^ M aldosterone or vehicle treatment. After 30 minutes, total protein was collected and the expression level of the indicated protein was measured by Western blot with specific antibodies. (C) NF-κB was a prerequisite transcriptional factor in aldosterone-mediated galectin-3 expression. THP-1 cells were transfected with NF-κB decoy ODN, AP-1 decoy ODN and serum starved for 24 h, then treated with 10^−6^ M aldosterone for another 24 h. ^*^ *P*<0.05. (D) Aldosterone activated NF-κB via MR/PI3K/Akt signaling. THP-1 cells were serum-starved for 24 h and then pre-treated with 10^−7^ M of spironolactone, LY294002 (50 µg/ml) or BAY117082 (10 µM) for 1 h before 10^−6^ M aldosterone treatment. Nuclear and cytosolic proteins were collected after 1 h, and the level of the NF-κB p65 subunit was determined by Western blotting. MR =  mineralocorticoid receptor. nuclear (N) p65 and cytosol (C) p65.

**Figure 6:**
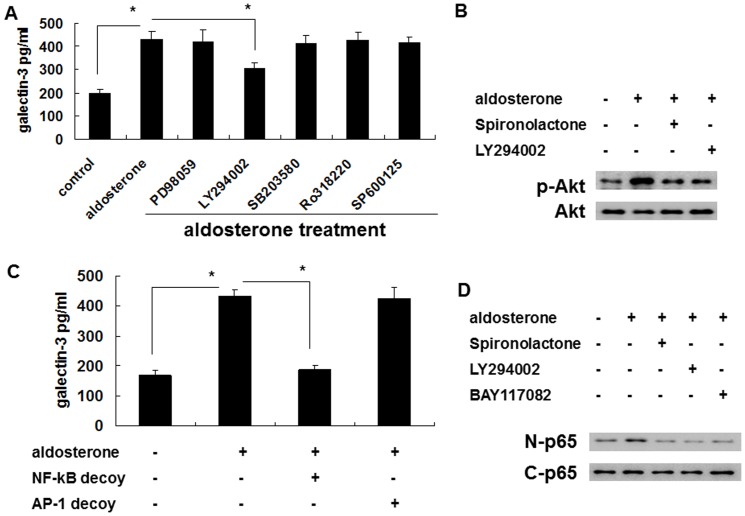
Aldosterone induced galectin-3 expression in Raw 264.7 cells via a MR/PI3K/Akt/NF-kB signaling pathway. (A) Aldosterone induced galectin-3 expression in Raw 264.7 cells via the PI3K/Akt signaling pathway. Raw 264.7 cells were serum-starved for 24 h and then pre-treated with PD98059 (50 µg/ml), LY294002 (50 µg/ml), SB203580 (5 µg/ml), Ro318220 (5 nM), and SP600125 (10 µM) for 1 h prior to 5×10^−7^ M aldosterone or vehicle treatment for 24 h. (B) Aldosterone induced Akt phosphorylation via MR-mediated signaling. Raw 264.7 cells were serum-starved for 24 h, then pre-treated with different chemical inhibitors for 1 h prior to 5×10^−7^ M aldosterone or vehicle treatment. After 30 minutes, total protein was collected and the expression level of the indicated protein was measured by Western blot with specific antibodies. (C) NF-κB was a prerequisite transcriptional factor in aldosterone-mediated galectin-3 expression. Raw 264.7 cells were transfected with NF-κB decoy ODN, AP-1 decoy ODN and serum starved for 24 h, then treated with 5×10^−7^ M aldosterone for another 24 h. ^*^ *P*<0.05. (D) Aldosterone activated NF-κB via MR/PI3K/Akt signaling. Raw 264.7 cells were serum-starved for 24 h and then pre-treated with 10^−7^ M of spironolactone, LY294002 (50 µg/ml) or BAY117082 (10 µM) for 1 h before 5×10^−7^ M aldosterone treatment. Nuclear and cytosolic proteins were collected after 1 h, and the level of the NF-κB p65 subunit was determined by Western blotting. MR =  mineralocorticoid receptor; nuclear (N) p65 and cytosol (C) p65.

The induced secretion was blocked by LY294002, but not by PD98059, SB203580, Ro318220, or SP600125. This suggested that the induced secretion occurred via the PI3K/Akt pathway (Figure 5A, 6A). To confirm that the PI3K/Akt pathway was involved in the aldosterone signaling, spironolactone was used to evaluate whether or not MRs were upstream of the aldosterone-induced Akt phosphorylation. The result of p-Akt Western blotting revealed that the MRs were indeed upstream of the aldosterone-induced Akt phosphorylation (Figure 5B, 6B).

In transcriptional factor analysis, induction of galectin-3 secretion was blocked by the decoy ODN of NF-κB but not the decoy ODN of AP-1. This suggested that NF-κB is an important transcriptional factor in the secretion pathway (Figure 5C, 6C). Spironolactone and LY294002 were used to evaluate the roles of MRs and PI3K/Akt in the aldosterone-induced NF-κB activation. The results of NF-κB nuclear and cytosol Western blotting showed that MRs and PI3K/Akt were upstream of the aldosterone-induced NF-κB activation (Figure 5D, 6D).

### Aldosterone-induced galectin-3 expression plays a critical role in enhancing fibrosis-related factor expression in fibroblasts

**Figure 7:**
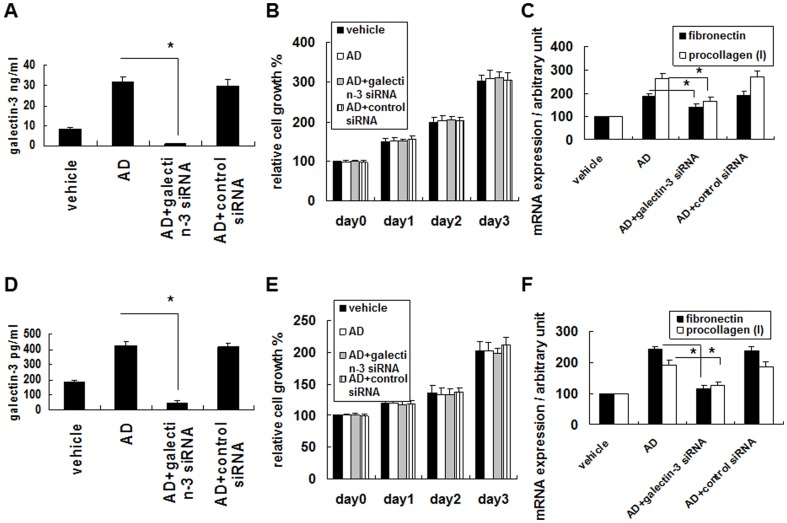
Aldosterone-induced galectin-3 in THP-1 and RAW 264.7 cells enhanced fibrosis-related factor in fibroblasts. (A) Effect of galectin-3 siRNA treatment on inhibiting aldosterone-induced galectin-3 expression. THP-1 cells were pretreated with galectin-3 siRNA or control siRNA for 24 h and then serum-starved for 24 h prior to 10^−6^ M aldosterone or vehicle treatment for 24 h. The conditional medium was collected, and the level of galectin-3 was detected by enzyme immunoassay. (B) Effect of conditional medium from aldosterone-treated THP-1 cells on fibroblast cell growth. The conditional medium was collected and cultured with the human fibroblast cell line 293T. Cell growth was determined by MTT assay. Data were recorded as relative cell growth in the indicated conditions with the vehicle-treated conditional medium in lane 1 defined as 100%. *, *P*<0.05 (n = 5). (C) Effect of conditional medium from aldosterone-treated THP-1 cells on fibronectin and procollagen (I) mRNA expressions. Conditional medium as described in (A) was used to treat 293T cells. After 24 hours, the expressions of fibronectin and procollagen (I) mRNA were determined by real-time quantitative RT-PCR. Data were compared between the indicated groups. A paired Student's *t*-test was used to evaluate statistically significant differences between the indicated groups. ^*^ *P*<0.05. (D) Effect of galectin-3 siRNA treatment on inhibiting aldosterone-induced galectin-3 expression. Raw 264.7 cells were pretreated with galectin-3 siRNA or control siRNA for 24 h, and then serum-starved for 24 h prior to 10^−6^ M aldosterone or vehicle treatment for 24 h. The conditional medium was collected, and the level of aldosterone was detected by enzyme immunoassay. (E) Effect of conditional medium from aldosterone-treated RAW 264.7 cells on fibroblast cell growth. The conditional medium was collected and cultured with the mouse fibroblast cell line NIH/3T3. The cell growth was determined by MTT assay. Data were recorded as relative cell growth in the indicated conditions with the vehicle-treated conditional medium in lane 1 defined as 100%. *, *P*<0.05 (n = 5). (F) Effect of conditional medium from aldosterone-treated RAW 264.7 cells on fibronectin and procollagen (I) mRNA expressions. Conditional medium as described in (E) was used to treat NIH/3T3 cells. After 24 h, the expressions of fibronectin and procollagen (I) mRNA were determined by real-time quantitative RT-PCR. Data were compared between the indicated groups. A paired Student's *t*-test was used to evaluate statistically significant differences between the indicated groups. *P<0.05. AD =  aldosterone.

To evaluate the possible effect of aldosterone-induced galectin-3 expression on fibrosis, THP-1 and RAW 264.7 cells were pretreated with galectin-3 siRNA or control siRNA in order to knockdown the galectin-3 expression prior to aldosterone stimulation. The conditioned medium was collected and cultured with the human fibroblast cell line 293T. The aldosterone-induced galectin-3 expression was strongly suppressed by galectin-3 siRNA (Figure 7A, 7D), however there was no significant effect of aldosterone-induced galectin-3 on cell growth of the 293T or NIH/3T3 cells (Figure 7B, 7E). Meanwhile, the effect of the conditioned medium on the expressions of fibronectin and procollagen (I) mRNA was determined, and the results revealed that aldosterone-induced galectin-3 significantly increased the expressions of fibronectin and procollagen (I) mRNA of the 293T and NIH/3T3 cells (Figure 7C, 7F).

### Aldosterone induced galectin-3 expression in vivo

**Figure 8:**
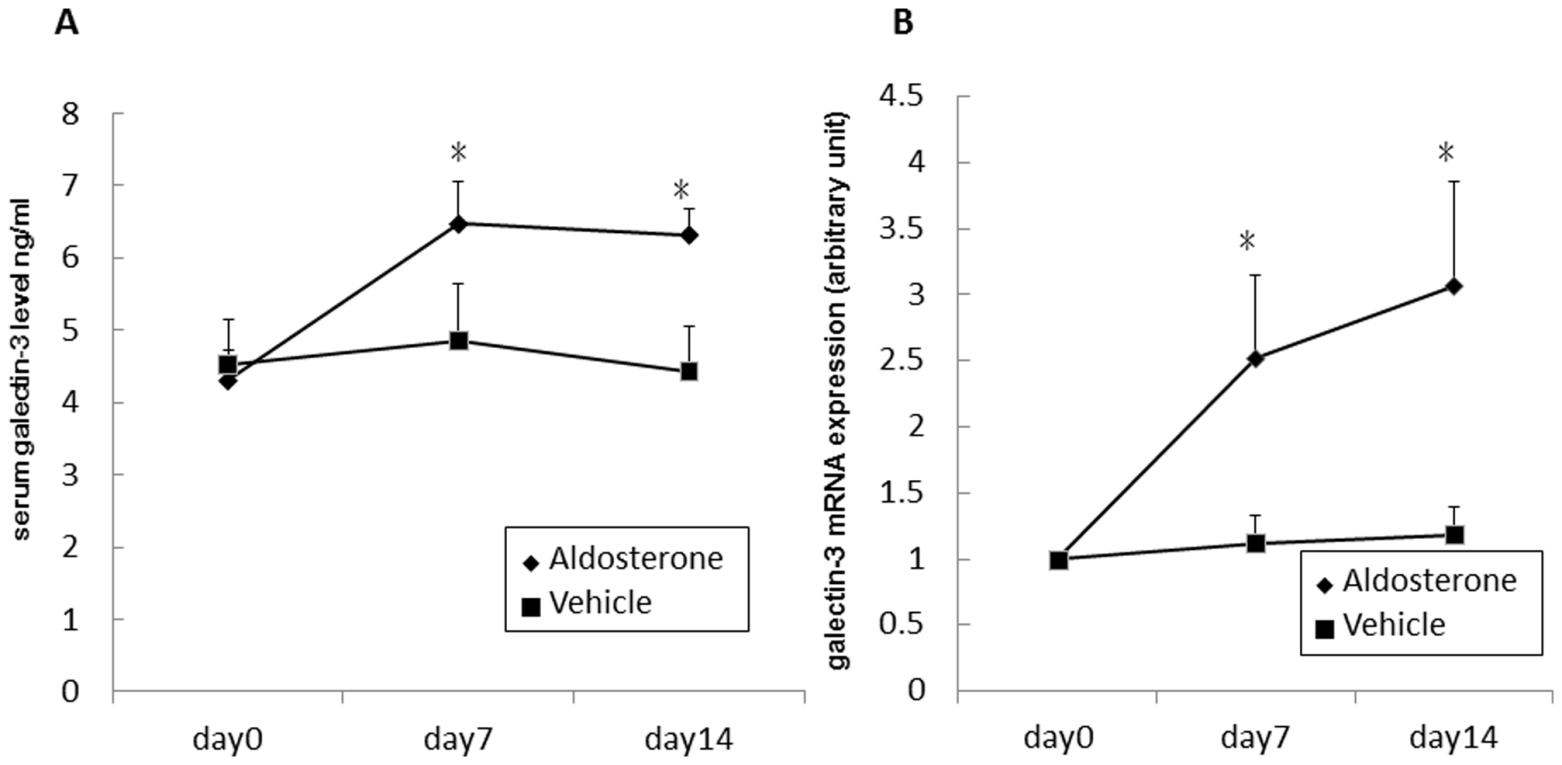
Aldosterone induced galectin-3 expression in vivo. ICR wild-type male mice were implanted subcutaneously with a 20-d continuous aldosterone release pellet. Mice whole blood was collected on days 0, 7, and 14. (A) Serum level of galectin-3 was detected by enzyme immunoassay. (B) Total RNA was purified from PBMCs. Galectin-3 mRNA expression was determined by quantitative RT-PCR. ^*^P<0.05. PBMCs  =  peripheral blood mononuclear cells.

To confirm the effect of aldosterone-induced galectin-3 expression in vivo, ICR wild-type male mice were implanted subcutaneously with a 20-d continuous aldosterone release pellet, and serum galectin-3 levels were measured. Galectin-3 mRNA was analyzed from total RNA purified from peripheral blood mononuclear cells. The results showed that both were significantly increased in the aldosterone pellet implanted mice on days 7 and 14 (Figure 8a, 8b).

### Patient characteristics

Sixteen EH patients and 7 APA patients who did not take spironolactone and received adrenalectomy within 6 months were enrolled in this study. The clinical data are shown in Table 1. The APA patients had significantly higher PAC, ARR, log PAC, log ARR, and lower PRA than the patients with EH. Moreover, the APA patients had a significantly higher plasma galectin-3 level than the EH patients (2.0±0.9 vs. 1.3±0.7 ng/ml, p = 0.039). The plasma galectin-3 level was borderline significantly correlated with log PAC (r = −0.365, p = 0.087), but not log PRA, log ARR, systolic blood pressure, or diastolic blood pressure (all p>0.10).

**Table 1:** Clinical data of the patients.

Patient characteristics	Group 1 (n = 7)	Group 2 (n = 16)	*p* value
Age, years	49±8	53±7	0.219
Male sex	2 (29)	3 (19)	0.621
Hypertension history, years	4.8±5.8	4.5±4.2.	0.900
Body weight, kg	61±11	60±10	0.768
Body height, cm	160±9	161±8	0.830
Creatinine	0.8±0.2	0.9±0.2	0.246
SBP, mmHg	150±22	145±18	0.523
DBP, mmHg	94±13	87±10	0.180
Potassium, mmol/L	3.6±0.7	4.1±0.3	0.122
PAC, ng/dL	77±42	26±19	0.019
PRA, ng/mL/h	0.6±0.8	7.0±8.3	0.008
ARR	2638±4310	15±18	0.158
Log PAC	1.8±0.3	1.4±0.2	0.001
Log PRA	−0.8±0.9	0.5±0.5	<0.001
Log ARR	2.6±1.1	0.8±0.6	0.004
Number of anti-hypertensive agent	2.1±1.3	1.6±1.0	0.252
Galectin-3, ng/ml	2.1±0.9	1.2±0.7	0.020

Values are mean ± SD or number (percentage). APA  =  aldosterone producing adenoma; EH =  essential hypertension; SBP  =  systolic blood pressure; DBP  =  diastolic blood pressure; PAC  =  plasma aldosterone concentration; PRA  =  plasma renin activity; ARR  =  aldosterone-renin ratio.

### Post-adrenalectomy follow-up

One year after adrenalectomy, 3 APA patients (38%) were cured of hypertension. In serological studies, log PAC significantly decreased and log PRA significantly increased (log PAC 1.8±0.3 to 1.5±0.1; log PRA −0.8±0.9 to 0.2±0.8, both p<0.05). The plasma galectin-3 level was also significantly decreased (from 2.1±0.9 to 1.2±0.7 ng/ml, p = 0.020).

## Discussion

**Figure 9:**
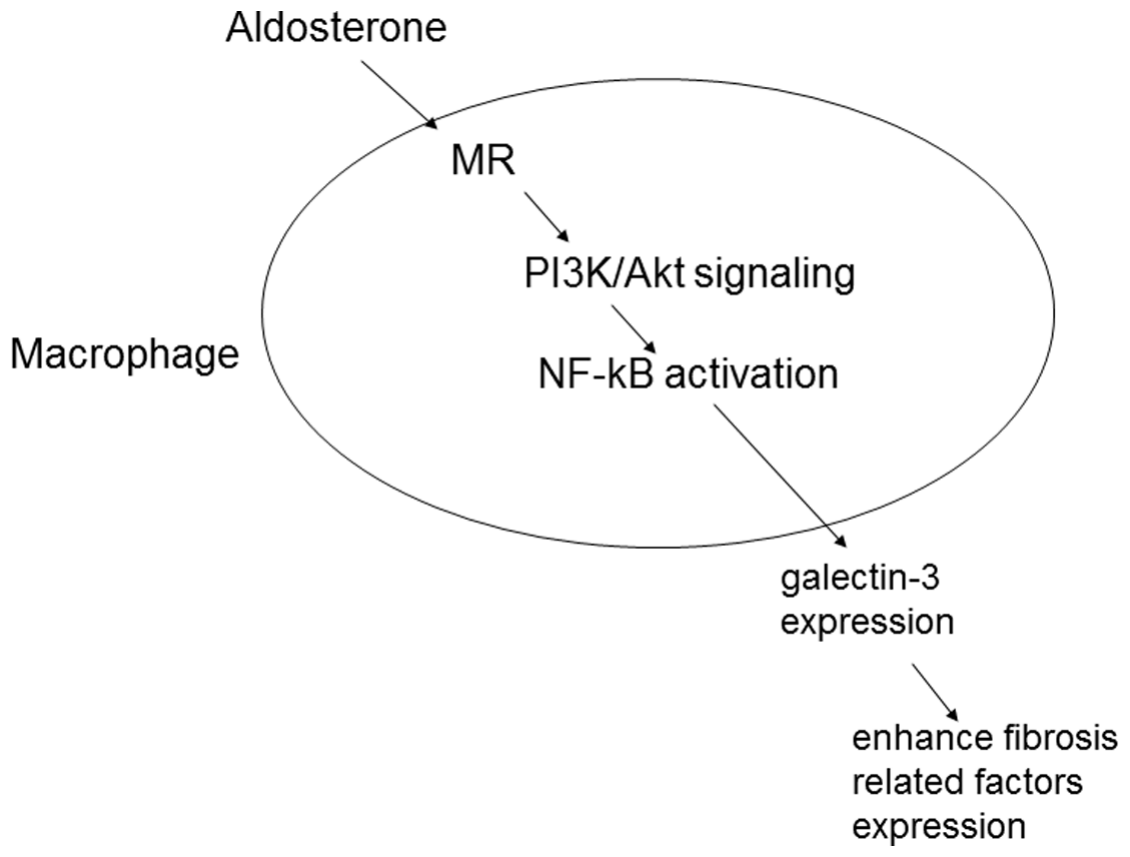
Schematic signaling and possible function of aldosterone-induced galectin-3 expression in macrophages. Through a MR/PI3K/Akt/NF-kB signaling pathway, aldosterone-induced galectin-3 expression enhanced fibrosis-related factor expression in fibroblasts.

The results of this study demonstrate a novel signal transduction pathway of galectin-3 secretion by aldosterone in macrophage cell lines, and the impact of galectin-3 on collagen formation (Figure 9). Furthermore, we found that aldosterone could induce galectin-3 secretion by macrophages in a mouse model, and an association between aldosterone and galectin-3 in a preliminary clinical study.

Aldosterone can induce myocardial and vascular fibrosis, and aldosterone infusion with high salt intake has been shown to induce cardiac hypertrophy, myocardial interstitial fibrosis and scarring in the right and left ventricles[4]. Furthermore, aldosterone infusion has also been shown to induce increased interstitial fibrosis in the atria and pulmonary arteries[29] which is not influenced by systemic blood pressure. In a rat model, infra-renal aorta banding was found to induce hypertension and cardiac hypertrophy, but not to increase myocardial fibrosis[30].

The proposed mechanism of aldosterone-induced myocardial fibrosis has changed in recent years. Aldosterone-induced macrophage activation and low grade inflammation have been proven to play important roles in cardiac fibrosis[2]. Furthermore, Rickard et al demonstrated that specific deletion of macrophage MRs prevented cardiac fibrosis induced by deoxycorticosterone in a mouse model[11].

Galectin-3 has also been reported to play an important role in failing hearts and HF-related cardiac fibrosis. Sharma et al reported that galectin-3 was one of the most robustly over-expressed mediators in failing hearts in microarray studies[12]. Ochieng et al reported that galectin-3 interacts with various ligands in the extracellular matrix (ECM), including laminin, synexin, integrins, and collagen[31], and Sharma et al suggested that activated cardiac macrophages produce galectin-3 and recognize the galectin-3 binding sites in cardiac fibroblasts and the ECM [12]. Sharma et al also showed that intrapericardial infusion of recombinant galectin-3 in healthy rats can result in a depressed LV ejection fraction and increased collagen volume fraction in the myocardium [12]. Further, they showed that cardiac macrophages were activated in failure-prone rat hearts, and that the expression of galectin-3 was increased and co-localized with activated myocardial macrophages[12]. Sharma et al also found galectin-3 binding sites in the ECM, and cardiac fibroblasts and galectin-3-induced cardiac fibroblast proliferation, collagen production, and cardiac dysfunction in rats. These findings support the relationship between inflammation and fibrosis, and provide a new insight into the mechanism in ECM turnover in HF. Further, the findings imply that galectin-3 may play an important role in the regulation of myocardial fibrosis and pathogenesis of HF. In our previous study on HF patients, serum galectin-3 was found to be significantly correlated with serum markers of the ECM, and that ECM turnover is an essential process in LV remodeling and interstitial fibrosis[15].

Galectin-3 is also a crucial factor in hyperaldosteronism in combination with hypertension-induced cardiac fibrosis[13] and vascular fibrosis[20]. A previous study reported that hyperaldosteronism combined with hypertension stimulated macrophage infiltration in the heart, and enhanced the mRNA level of galectin-3 [13]. Furthermore, blocking the MRs with eplerenone could reverse the effect of aldosterone on cardiac fibrosis and cardiac galectin-3 mRNA production. These findings not only demonstrate the importance of aldosterone on cardiac fibrosis, but also suggest the possible effect of galectin-3 secretion by aldosterone. Our study provides further evidence of galectin-3 secretion by aldosterone stimulation and the possible signal transduction pathway of the process. In addition, this is the first study to provide human evidence of increased galectin-3 secretion in patients with hyperaldosteronism and the effect of adrenalectomy on galectin-3 level.

In the current study, aldosterone-induced galectin-3 secretion started from 10^−7^ M (1*10^−7^ M in the THP-1 cell study and 2.5*10^−7^ M in the RAW 264.7 cell study). A normal human PAC is around 0.2–1.0 *10^−9^ M, and the PAC in patients with PA is 2 to 10-fold higher than normal subjects. However, PA is a chronic disease. To investigate the long-term effects of aldosterone exposure we used a relatively high aldosterone concentration in the cell and animal studies.

Calvier et al detected intracellular galectin-3 mRNA and protein formation after aldosterone stimulation in vascular smooth muscle cells, and concluded that galectin-3 is essential in aldosterone-induced collagen production in vascular smooth muscle cells[32]. The aldosterone effect was found to be significant from 10^−9^ M in that study, which is lower than the dosage used in the current study. However, there are several differences between the two studies. First, Calvier et al detected intracellular galectin-3 mRNA and protein, and as a result, vascular smooth muscle cells acted as both producers and effectors of galectin-3. This result, however, fails to explain the findings of the study by Rickard et al[11], in which deletion of macrophage MRs was found to protect the process of cardiac fibrosis, implying that macrophage MRs are involved in the formation of cardiac fibrosis. Second, we detected the secretion of galectin-3 from macrophages in our study, whereas Calvier et al detected intracellular galectin-3 mRNA and protein, and it would be easier to detect changes in galectin-3 level intracellularly under the stimulation of a lower aldosterone concentration. Third, the tissue concentration of aldosterone is different from the plasma concentration. In the process of aldosterone-induced myocardial fibrosis, macrophages may be activated in cardiac tissue rather than in peripheral blood. In a previous study, the tissue aldosterone concentration was reported to be 6 pg/mg in atrial tissue[33]. However, the tissue concentration is measured from total tissue extract diluted in a fixed volume. Therefore, PAC and tissue aldosterone concentration cannot be compared directly.

There are some limitations to this study. First, this study focused on the process of aldosterone-induced galectin-3 secretion, and the relationship between aldosterone-induced galectin-3 secretion and fibrosis was only studied in cellular experiments. Further animal and human studies related to this topic are needed to obtain a full understanding of the mechanism. Second, the number of patients in the clinical study was small, and the clinical findings of this study should be confirmed by a larger clinical study. Further, the correlation between plasma aldosterone and galectin-3 concentration was borderline significant. This relationship should also be studied in a larger clinical study.

## Conclusion

Aldosterone could induce galectin-3 secretion in macrophage cell lines and further enhance collagen formation by fibroblasts. The pathway was through mineralocorticoid receptors, and the PI3K/Akt and NF-κB transcription pathways. Galectin-3 secretion induced by aldosterone was further confirmed by an aldosterone-infusing mouse model. The association between aldosterone and galectin-3 was also found in a preliminary clinical study.
